# Periosteum and fascia lata: Are they so different?

**DOI:** 10.3389/fbioe.2022.944828

**Published:** 2022-10-19

**Authors:** Julie Manon, Robin Evrard, Louis Maistriaux, Lies Fievé, Ugo Heller, Delphine Magnin, Jean Boisson, Natacha Kadlub, Thomas Schubert, Benoît Lengelé, Catherine Behets, Olivier Cornu

**Affiliations:** ^1^ Neuromusculoskeletal Lab (NMSK), Institut de Recherche Expérimentale et Clinique (IREC), UCLouvain, Brussels, Belgium; ^2^ Morphology Lab (MORF), IREC, UCLouvain, Brussels, Belgium; ^3^ Transplantation and Experimental Surgery Lab (CHEX), IREC, UCLouvain, Brussels, Belgium; ^4^ Centre de Thérapie Cellulaire et Tissulaire Locomoteur, Cliniques Universitaires Saint-Luc, Brussels, Belgium; ^5^ APHP, Necker Enfants Malades, Unit of Maxillofacial Surgery and Plastic Surgery, Paris, France; ^6^ Department of Mechanical Engineering, Ecole Nationale Supérieure de Techniques Avancées (ENSTA) de Paris, Institut des Sciences de la Mécanique et Applications Industrielles (IMSIA), Paris, France; ^7^ Bio- and Soft Matter (BSMA), Institute of Condensed Matter and Nanosciences (IMCN), Louvain-la-Neuve, Belgium

**Keywords:** periosteum, fascia lata, type 1 collagen membrane, tissular properties, tissular composition, tissue engineering, fracture healing

## Abstract

**Introduction:** The human fascia lata (HFL) is used widely in reconstructive surgery in indications other than fracture repair. The goal of this study was to compare microscopic, molecular, and mechanical properties of HFL and periosteum (HP) from a bone tissue engineering perspective.

**Material and Methods:** Cadaveric HP and HFL (*N* = 4 each) microscopic morphology was characterized using histology and immunohistochemistry (IHC), and the extracellular matrix (ECM) ultrastructure assessed by means of scanning electron microscopy (SEM). DNA, collagen, elastin, glycosaminoglycans, major histocompatibility complex Type 1, and bone morphogenetic protein (BMP) contents were quantified. HP (*N* = 6) and HFL (*N* = 11) were submitted to stretch tests.

**Results:** Histology and IHC highlighted similarities (Type I collagen fibers and two-layer organization) but also differences (fiber thickness and compaction and cell type) between both tissues, as confirmed using SEM. The collagen content was statistically higher in HFL than HP (735 vs. 160.2 μg/mg dry weight, respectively, *p* < 0.0001). On the contrary, DNA content was lower in HFL than HP (404.75 vs. 1,102.2 μg/mg dry weight, respectively, *p* = 0.0032), as was the immunogenic potential (*p* = 0.0033). BMP-2 and BMP-7 contents did not differ between both tissues (*p* = 0.132 and *p* = 0.699, respectively). HFL supported a significantly higher tension stress than HP.

**Conclusion:** HP and HFL display morphological differences, despite their similar molecular ECM components. The stronger stretching resistance of HFL can specifically be explained by its higher collagen content. However, HFL contains many fewer cells and is less immunogenic than HP, as latter is rich in periosteal stem cells. In conclusion, HFL is likely suitable to replace HP architecture to confer a guide for bone consolidation, with an absence of osteogenicity. This study could pave the way to a bio-engineered periosteum built from HFL.

## 1 Introduction

Periosteum plays a major role in long bone fracture healing and transverse bone growth (Orwoll, 2003; Dwek, 2010; Chen et al., 2022). This richly vascularized membrane surrounding bone is made up of two layers. The outer one, the fibrous or sterile layer, is mainly composed of dense connective tissue, i.e., Type 1 collagen fibers and fibroblasts. The inner layer, the cambial or fertile layer, is in close contact with the cortical bone, and it participates in bone modeling, as well as initiating the fracture callus, owing to the primordial attendance of periosteal mesenchymal stem cells (Orwoll, 2003; Dwek, 2010; Evans et al., 2013).

However, in the challenging treatment of a critical size bone defect (CSBD) following trauma, tumor resection, infection, or congenital defect, the periosteum can be missing and thus no longer able to contribute to bone healing. This research field has grown rapidly since the 2000s, with multiple new therapeutic options made available, such as bone allograft, vascular autograft, surgical bone transport technique, synthetic implants, and new engineered periosteum scaffold, leading to some improvements but often being burdened by complications nevertheless (Delloye et al., 2014; Chen et al., 2015; Moore et al., 2016; Yu et al., 2020; Dalisson et al., 2021; Chen et al., 2022). Among previous approaches, Masquelet’s induced membrane (Masquelet, 2020) reaches nearly all (4/5) of the diamond concept conditions (osteoconductive matrix, osteogenic cells, osteoinductive mediators, mechanical stability, and vascularization), leading to an 89% consolidation rate (Andrzejowski and Giannoudis, 2019). However, it requires a two-stage surgery to debride and insert a cemented spacer prior and delicately open the newly formed membrane and replace the spacer with a bone autograft. This induced membrane plays the role of a well vascularized periosteum-like tissue, without exactly mimicking it, due to the lack of mesenchymal stem cells and the specific periosteal microarchitecture. This tubing sheet also plays a role in guided bone regeneration and barrier membrane. This concept has times been reported in the literature as being designed to conduct and promote bone healing, as well as preventing soft tissue invasion (Yu et al., 2015). The development of an off-the-shelf induced membrane that could play the same role but in a one-stage surgery may emerge as a solution that could reduce the time and costs involved in this challenging surgical treatment.

Most authors who seek to develop a new scaffold based on tissue engineering techniques usually go straight to their target, while bypassing fundamental tissue knowledge. The iliotibial tract, which constitutes a thickening of the fascia lata of human thigh, is a biological sheet that is already used in other reconstructive surgeries, such as duraplasty (Finn et al., 2011), colpopexy (Bock et al., 2021), and abdominal wall reconstruction (Song et al., 2018) instead of an artificial mesh, or for head and neck reconstruction as vascularized flap (Janik et al., 2020). In orthopedics, the human fascia lata (HFL) has already been employed to repair rotator cuff tear (Matthewson et al., 2020), in the “anchovy” interpositional arthroplasty of the hallux metatarsophalangeal joint (Watson et al., 2019), to reconstruct patellar ligament (Sapino et al., 2019) or after anterior cruciate ligament rupture (Ferretti et al., 2017); nevertheless, it has never been considered for CSBD treatment. The fascia lata has already been used to enhance the stability and support strength of other soft tissues, such as the periosteum (Yu et al., 2015), although the HFL has never been directly compared to the human periosteum (HP) from a tissue engineering point of view. The goal of this study was to compare the HFL microscopic, molecular, and mechanical characteristics/properties with those of HP in order to explore its potential use as a periosteal-like scaffold in the treatment of CSBD.

## 2 Materials and methods

### 2.1 Human tissue harvesting

Femoral HP and HFL were obtained from cadaveric donors (one woman and four men; average age: 91.3 ± 1.8 years) who were received at the Human Anatomy Department of UCLouvain (IRB00008535, Brussels, Belgium), following local ethics committee authorization (Ref 2021-30AOU-356 approved on 13 September 2021). For the mechanical tests, independent tissues were collected from other donors (11 HFL from three women and three men, average age: 88.8 ± 3.2 years; six HP from two women and three men, average age: 86.4 ± 6.0 years). These bodies were provided by the Ecole de Chirurgie de Paris (Agence Générale des Equipements et Produits de Santé [AGEPS], Assistance Publique Hôpitaux de Paris [APHP]). Permission to perform the study using cadaveric specimens was obtained from the institutional review board (Ecole de Chirurgie, AGEPS, APHP, IRB00011591, Paris, France). All cadaveric subjects had previously provided their consent for their body being employed for medical research.

All the samples were harvested from the thigh using a longitudinal incision from the anterosuperior iliac spine to the upper border of the patella. The HFL was first cautiously dissected and sectioned from the median line to the lateral lip of linea aspera. Then, the quadriceps femoris was carefully elevated, and the HP was harvested using a rugin following a circumferential incision above the femoral condyles and below the lesser trochanter. Both tissues were divided into several samples that were differently processed in the following analyses.

### 2.2 Histological analysis

Micromorphological analyses were explored using classical histology. In each tissue from four donors, three 1 cm^2^ samples were fixed in formaldehyde 4% (VWR, 9713.9010), embedded in paraffin, and sectioned in 5 µm thick transverse or longitudinal slices, which were stained using hematoxylin and eosin (H&E), Masson’s trichrome (MT), Sirius red (SR), and alcian blue (AB), following the manufacturers’ protocols. In order to examine deoxyribonucleic acid (DNA), sections were also stained with 2'-(4-ethoxyphenyl)-5-(4-methyl-1-piperazinyl)-2,5′-bi-1H-benzimidazole trihydrochloride (Hoechst, 1:5,000; Life Technologies, H3570). All sections were captured with a slide scanner (SCN400, Leica Microsystems, Wetzlar, Germany) or visualized using a fluorescence microscope (AxioImager Z1, Zeiss, Oberkochen, Germany) at a 40-fold magnification. The images were then annotated with ImageScope software (Aperio ImageScope, v12.4.3.5008, Leica Biosystems). This latter software was also used to measure the tissue thickness of the MT-stained sections. The HP cambial layer and HFL transverse layer were both the thinnest layers, termed Layer 1 (L1). The HP fibrous layer and HFL longitudinal layer were both the thickest layers, termed Layer 2 suppress = (L2). The percentage of the thinnest layer, which is useful to bear mesenchymal stem cells, was calculated for each sample, as follows:

$$Layer\;percentage\;\left(\%\right)=\frac{L1\;thickness}{Total\;thickness}\;x\;100$$

### 2.3 Immunohistochemistry (IHC) analysis

Paraffin-embedded slices were handled for immunohistochemical detection of collagen I, major histocompatibility complex-1 (MHC-1), and elastin. Endogenous peroxidase was inactivated with 3% hydrogen peroxide in methanol. Antigen retrieval was achieved by a combination of tris-ethylenediaminetetraacetic acid (EDTA) buffer (Tris, Merck, 1.08387.250; EDTA, Sigma, E5134) for 5 min and then proteinase K (1:1,000, Roche, 03115828001) for 20 min, both at 37°C in the incubator. Unspecific antigens were blocked at room temperature (RT) by a solution of 5% bovine serum albumin (BSA, Merck, 12659-500 GM) in 0.05% tris-buffered saline (TBS)/Triton (Tris, Merck, 1.08387.250; Triton, VWR, M143) over 1 h. The sections were incubated with rabbit primary antibodies, namely anti-collagen I (1:1,000, Abcam, ab34710), anti-MHC-1 (1:150, Abcam, ab134189), and anti-elastin (1:100, Novus biotechne, NB100-2076) at 4°C overnight, followed by peroxidase conjugated anti-rabbit secondary antibody (Envision, Dako, K4003) at 4°C for 1 h. The detection was achieved with 3,3′-diaminobenzidine (DAB) peroxidase substrate (Dako, K3468) at RT for 5 min. Hematoxylin was used as counterstaining. After mounting, the slices were scanned using the Leica slide scanner. The MHC-1 was quantified by QuPath Software (v0.3.0., University of Edinburgh) (Bankhead et al., 2017).

Fluorescent multiplex IHC (indirect immunofluorescence HRP-conjugated secondary antibody) with tyramide amplification signal was performed on paraffin-embedded slices in order to detect CD73, CD90, and CD105, which are three specific surface markers of mesenchymal stem cells, according to the International Society for Cellular Therapy (Horwitz et al., 2005; Dominici et al., 2006). The same initial steps as conventional IHC were executed and then followed by antigen retrieval, realized by citrate buffer (citric acid, Merck 1.00244.0500; Na3 citrate, Alfa Aesar A12274-500 gr) at pH 5.7 in the microwave. Unspecific antigens were blocked by a solution of 5% BSA (Karl Roth, 3854.3, Albumin fraction V) in TBS/Tween 20 (VWR, 663684B) at RT during 30 min. The slices were incubated with first mouse primary monoclonal antibody for CD105/Endoglin (1:200, Cell Signaling, #14606) at 4°C overnight. Sections were then washed using TBS/Tween three times for 3 min. The peroxidase conjugated anti-mouse secondary antibody (Envision, Dako, K4001) was then incubated at RT for 45 min. Signal was amplified by AlexaFluor Tyramide 647 (1:200, InvitroGen, B40958) at RT for 10 min in borate solution (boric acid, Sigma B6768-500gr; NaCl, VWR, 27810.295-1kg; H2O2 0.003%). After three new washes, the steps from antigen retrieval were repeated using successively rabbit primary monoclonal antibodies for NT5E/CD73 (1:300, Cell Signaling, #13160) and for Thy1/CD90 (1:100, Cell Signaling, #13801) and tyramides as amplifiers (AlexFluor Tyramide 555 (1:200, InvitroGen, B40955) and 488 (1:200, InvitroGen, B40953), respectively). Hoechst was used for counterstaining (1:1,000, Sigma, 14533-100 mg). After mounting, the slices were scanned using the fluorescent slide scanner Axioscan. A negative control, without the primary antibody, was always included to check for absent detection for both standard IHC and multiplex IHC.

### 2.4 Scanning electron microscopy (SEM)

The ultrastructure of the extracellular matrix (ECM) was assessed superficially by means of scanning electron microscopy (SEM). One sample from each of four donors and for each tissue was cut into 5 mm^2^ pieces and mounted on synthetic corks. These samples were fixed by immersion in 3% glutaraldehyde buffered with 0.1 M phosphate buffer at RT for 4 h. They were then rinsed three times using 0.1 M phosphate buffer during 10 min. For the first dehydration process, the specimens were dehydrated in a series of graded ethanol dilutions (30%, 50%, 70%, 80%, 90%, 95%, and 3 × 100%) and then dried in specimen bottles on a slow shaking plate with an equilibration step of 15 min each. After the first dehydration process, the samples were dried using Critical Point Dryer technique (CPD) (Balzers, CPD020). The samples were then mounted on stubs and coated with a 10 nm gold layer (Cressington sputter, 208 HR) to create a thin conductive layer allowing for minimizing degradation and drifting due to thermal expansion. At least 12 pictures of SEM images for each tissue and donor were produced using a field-emission SEM (JSM-7600F, Jeol Ltd. Akishima, Tokyo, Japan) and then analyzed.

### 2.5 Cellular and extracellular matrix components quantification

DNA, collagen, elastin, and glycosaminoglycan (GAG) contents were quantified to compare both tissue compositions. For each dosage, three random biopsies of each tissue from each donor (*N* = 4) were performed, resulting in 24 analyzed biopsies of 25, 20, 25, and 10 mg for DNA, collagen, GAG, and elastin quantification. All biopsies were freeze-dried and dry-weighted. DNA was extracted using the DNeasy® Blood & Tissue kit (Qiagen, Italy), which included an overnight tissue lysis by proteinase K in a water-bath at 56°C and successive passages through DNeasy columns with different buffers, and was dosed using the Quant-iT PicoGreen DNA assay kit (ThermoFisher Scientific). After adding the Quant-iT PicoGreen reagent to the sample and following incubation for 5 min, the fluorescence was read using the SpectraMax (SofMax Pro 6 software) (excitation: 480 nm/emission: 520 nm). The collagen content was extracted and measured by means of the Quickzyme Total Collagen Assay (Quickzyme, Leiden, Netherlands). The extraction consisted of an overnight tissue lysis by HCl 6M in a water-bath at 95°C, while the dosage ended by an absorbance reading at 570 nm of wavelength. The Blyscan Sulfated-GAG assay kit (Biocolor LTD., Carrickfergus, Northern Ireland) was used to quantify the GAG content of ECM, and it also consisted of an extraction and dosage step using dye and dissociation reagents, while the final absorbance was read at 630 nm of wavelength. The elastin dosage was achieved by means of the Fast Elastin assay kit (Biocolor LTD., Carrickfergus, Northern Ireland). This quantification was based on two extraction repetition, which was followed by the dosage phase with successive additions and incubations of precipitating, dye and dye dissociation reagents (absorbance wavelength: 510 nm). All kits were employed in line with each detailed manufacturer’s protocol. Plates were filled in duplicates and read three times. Taking mean of the three readings was followed by taking the mean of the duplicates before performing the final average quantification of the three biopsies so as to increase the precision. The final DNA concentration was expressed in ng/mg of dry weight, while ECM proteins concentration was expressed in µg/mg of dry weight.

### 2.6 Immunoblot

Bone morphogenetic proteins (BMP) are growth factors that likely exert a relevant osteogenic function; they were evaluated using immunoblot in order to evaluate intrinsic osteogenic potential of both tissues. For this purpose, a 50 mg biopsy of each tissue (*N* = 3) was chopped, lysed with a radioimmunoprecipitation assay (RIPA) buffer containing protease inhibitor cocktail and Pho-Stop before being melded using the Precellys Homogenizer (Bertin Technologies SAS, France) by consecutive cycles at 7200 rpm. The supernatant was collected and its protein concentration was determined using the Pierce^TM^ BCA Protein assay kit (ThermoFisher, 23227) following the manufacturer’s protocol. Thereafter, the RayBio C-Series – Human BMP related array 2 (RayBiotech, AAH-BMP-2–8) quantified BMP-11, -2, -4, -5, -6, -7, -8, and -9. To this end, 100 µg of proteins were incubated in pre-blocked membrane (Blocking Buffer for 30 min at RT) on a shaker plate at RT for 2.5 h, followed by a succession of washes and incubations of biotinylated antibody cocktail, as well as diluted horseradish peroxidase (HRP)-Streptavidin according to the manufacturer’s protocol. Different baths of detection buffers preceded the revelation on chromatographic films by chemiluminescence. Using Fiji software (ImageJ-win64) and based on previously described methods (Di Meglio et al., 2017), each BMP densitometry spot was subtracted from the background density and normalized to positive control and to the ratio of the total amount of proteins to the weight of each respective sample (expressed in µg of proteins/mg of tissue). Final data were expressed as the mean density of two detection spots for each BMP followed by the mean of all donors.

### 2.7 Mechanical tests

Tensile tests were applied to underscore differences in mechanical properties. HFL (*N* = 11) were cut into five strips of 2 cm width and 5 cm length (total of analyzed samples = 55), while HP (*N* = 6) were cut into one strip of 1 cm width and 5 cm length (total of analyzed samples = 6). Specimens were stored frozen and then thawed at RT 2 h before testing. To carry out uniaxial tensile tests, samples were placed between two custom-made jaws in order to prevent slipping. The specimen length was measured between the jaws, with its width and thickness measured in its center using a caliper.

Traction tests were performed with two different uniaxial elongation machines, consisting of a 34SC-5 single column (Instron Corp., Illinois Tool Works Inc., Glenview, IL, United States) with a 5 kN load cell (2519-5 KN series, Instron Corp., Illinois Tool Works Inc., Glenview, IL, United States) for HFL and a 3342 single column (Instron Corp., Illinois Tool Works Inc., Glenview, IL, United States) with a 100N load cell (2519-100N series, Instron Corp., Illinois Tool Works Inc., Glenview, IL, United States) for HP. Four successive tests were conducted. Before testing, a preconditioning process consisting of 10 consecutive cycles at a speed of 0.25 mm/s to a final deformation of 7% was applied. Immediately thereafter, two tensile tests were conducted at two different speeds (S1 = 0.25 mm/s and S2 = 0.5 mm/s) up a strain of 10%. A final traction procedure was completed at 0.25 mm/s until complete rupture of the sample, which was defined as the drop of at least 40% of the force. The force and displacement data were collected during these tests and transformed into stress and strain values according to the following formulas:

$$Stress\equiv \sigma =\frac{F}{S}$$

where σ is the axial stress in Pascal (Pa), F the force in Newton (N) registered by the force cell, and S the section of the specimen (m^2^).

$$Strain\equiv \varepsilon =\frac{∆l}{l}$$

where ε is the strain (%), ∆l the distance variation measured during the tensile test, and l the initial distance between the two jaws.

The stress-strain curves are divided into two regions, of which one displayed a low slope at a small deformation (ε≲0.5) and the other one a large slope at high deformation. The slope of the curve’s steep linear part was considered the apparent elastic modulus of the sample. These moduli were extracted from each stress-strain curve for each speed. The rupture stress corresponded to the maximum value observed during the last traction test (rupture test).

### 2.8 Statistical analysis

Statistical comparison between both tissues was carried out using GraphPad Prism 8.0.1 (GraphPad Software, San Diego, CA, United States) and SPSS software (V.27, IBM SPSS, Inc., Chicago, IL, United States). The normality of continuous variables was checked using a Shapiro-Wilk test and QQ plots. All variables following a Gaussian distribution were compared by means of a parametric unpaired T-test for the thickness, which was also applied for proteins or DNA comparisons. In other cases, non-parametric tests (Mann-Whitney) were used to compare BMP factors between HP and HFL. All tests were two-tailed. The difference was always considered statistically significant at a *p*-value of 0.05. The data shown in the graphics are presented as the mean and standard error of the mean (SEM).

## 3 Results

### 3.1 Macro- and micro-structure

**FIGURE 1:**
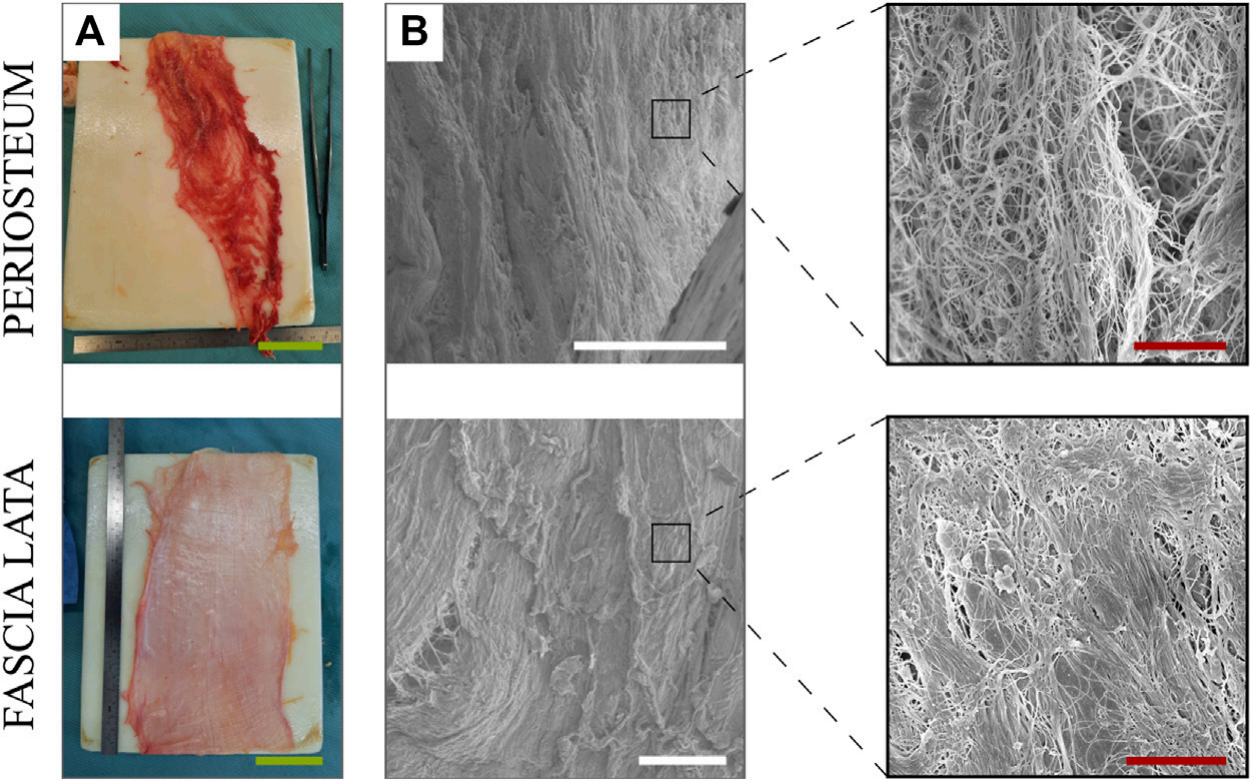
Macrostructure **(A)** and microstructure **(B)** of the periosteum (top) and fascia lata (bottom) (including zoom in), visualized under electron microscopy. Green scale bars: 5 cm. White scale bars: 100 µm. Red scale bars: 5 µm.

At first glance, the macrostructure of HP and HFL (Figure 1A) appeared to be quite different. Indeed, HP harvesting was more difficult and left holes in the tissular sheet, or it required preservation of muscle insertion, while HFL was taken in a single wide and long sheet with fewer holes created by the perforator vessels. However, the microstructure displayed some similarities under SEM analysis (Figure 1B). Both tissues were made of aligned fibers with a main orientation along the femoral axis. At a higher magnification, the density and organization of HFL fibers were more relevant. Density was characterized by a numerously higher compaction of fibers in a same area, while organization is about the higher fibers’ parallelism.

**FIGURE 2:**
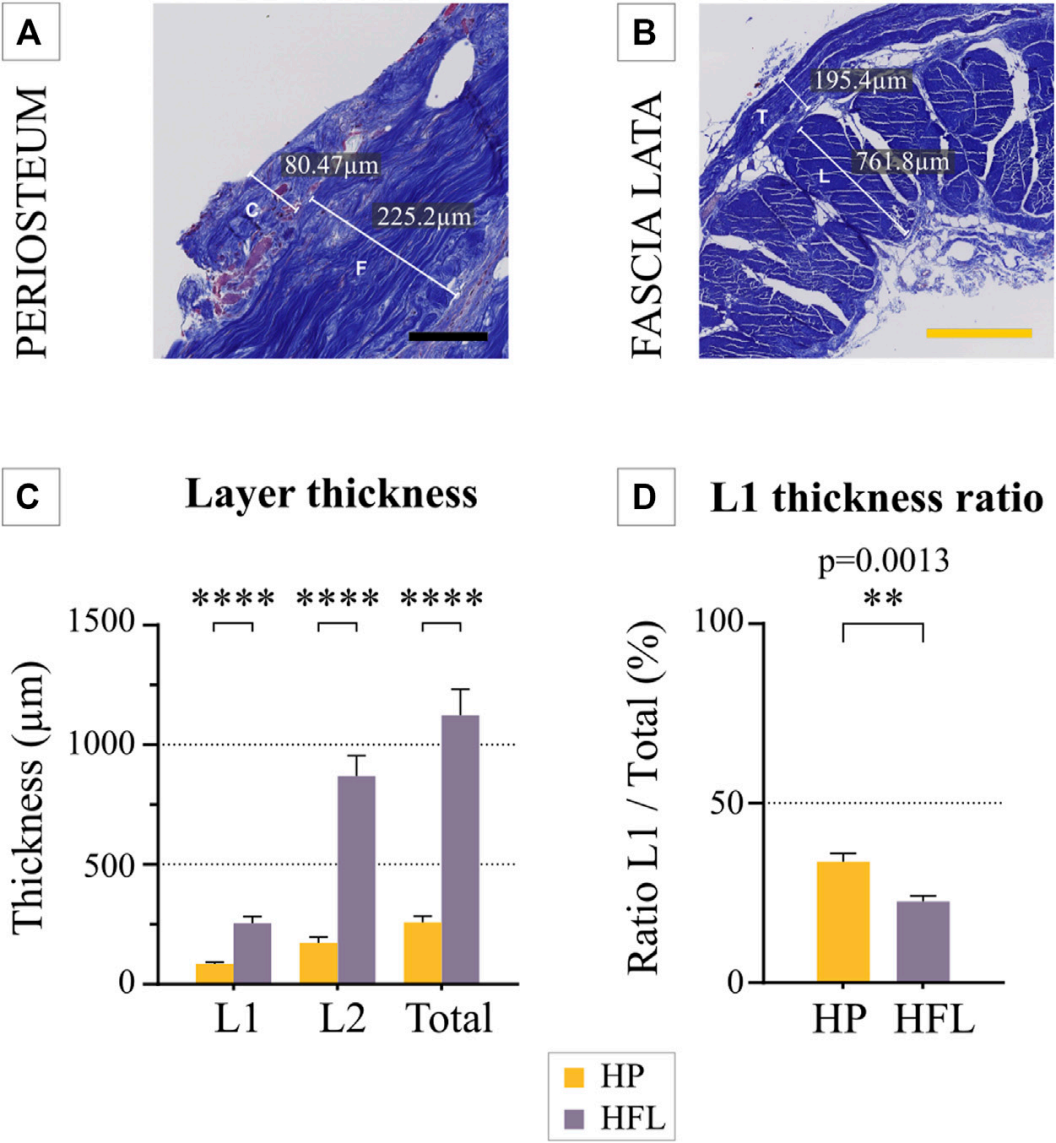
Example of a thickness measurement of the periosteum **(A)** and fascia lata **(B)**. The mean thickness of both tissues (*N* = 4 donors) was compared, along with their respective layers **(C)**, as well as the ratio of the thinnest layer to total thickness (*N* = 4 donors) **(D)**. C: cambial layer, F: fibrous layer, L: longitudinal layer, T: transverse layer, HP: human periosteum, HFL: human fascia lata, L1: cambial or transverse layer, L2: fibrous or longitudinal layer. Black scale bar: 100 µm. Yellow scale bar: 500 µm. ****p ≤ 0.0001.

Histological analysis highlighted the fibrous composition of both tissues, along with their bi-layered structure (Figure 2). Both tissues were built by two successive and well-defined layers. A dense connective tissue was present in the fibrous periosteal layer, with a loose connective tissue in the cambial one. HP was significantly thinner (258 ± 27 µm) than HFL (1,124 ± 108 µm). Similarly, HP cambial (85 ± 7 µm) and fibrous layers (173 ± 23 µm) were significantly thinner than HFL transverse (255 ± 29 µm) and longitudinal layers (869 ± 86 µm), respectively. However, the ratio of the thickness of the cambial layer to HP (34 ± 2%) was significantly greater than the ratio of the thickness of the transverse layer to the HFL (23 ± 2%).

### 3.2 Extracellular matrix compartment

The molecular components of ECM were identical for both tissues, containing Type 1 collagen fibers, GAG, and elastin.

#### 3.2.1 Collagen content

**FIGURE 3:**
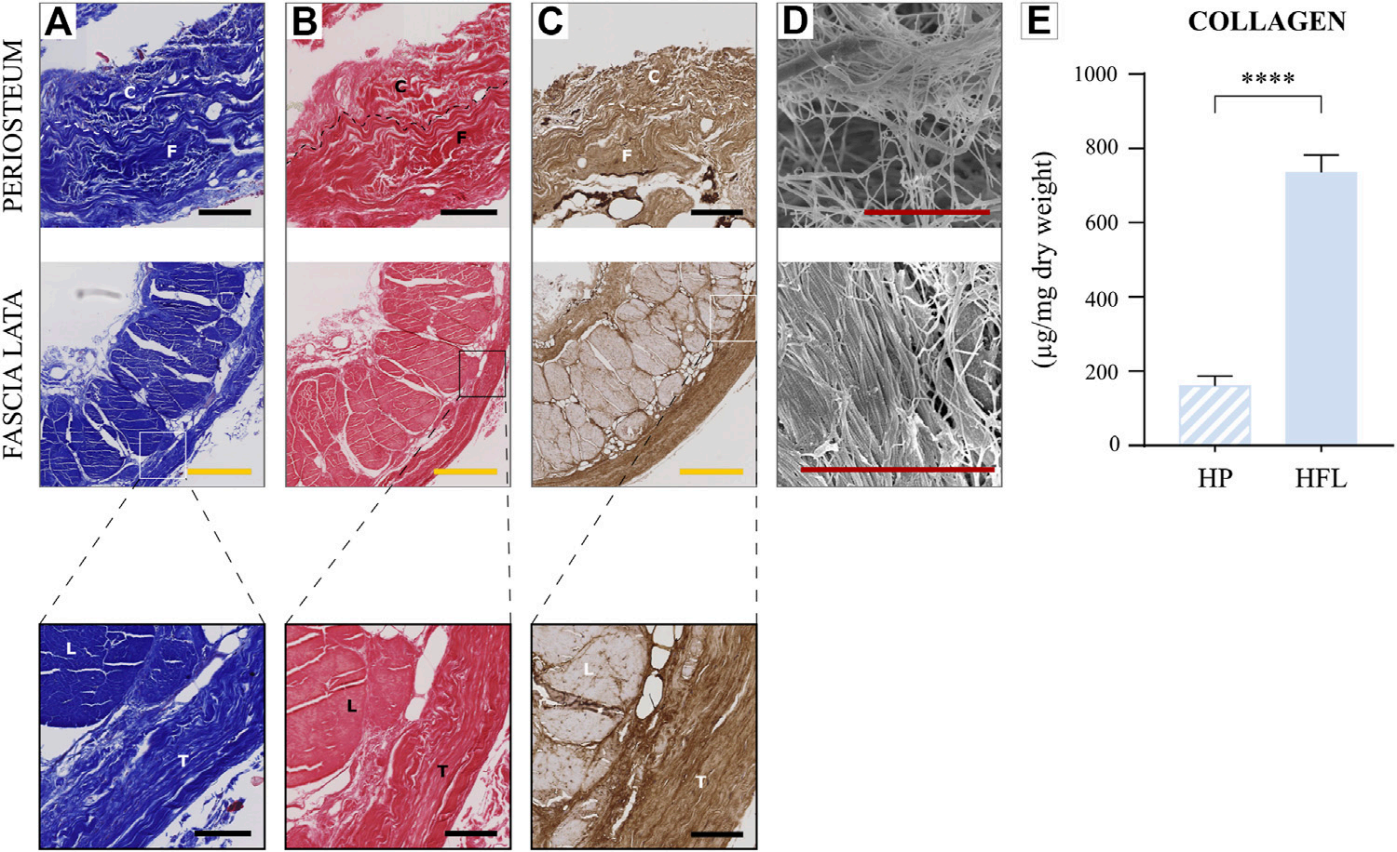
Comparison of HP (top) and HFL (bottom including zoom in) by Masson’s trichrome staining **(A)**, Sirius red staining **(B)**, immunohistochemistry for Type 1 collagen **(C)**, electron microscopy **(D)** and collagen quantification (N = 4 donors × three samples from each donor leading to *n* = 12 specimens analyzed for each tissue) **(E)**. C: cambial layer, F: fibrous layer, L: longitudinal layer, T: transverse layer, HP: human periosteum, HFL: human fascia lata. Black scale bars: 100 µm. Yellow scale bars: 500 µm. Red scale bars: 5 µm. **** *p* < 0.0001.

**FIGURE 4:**
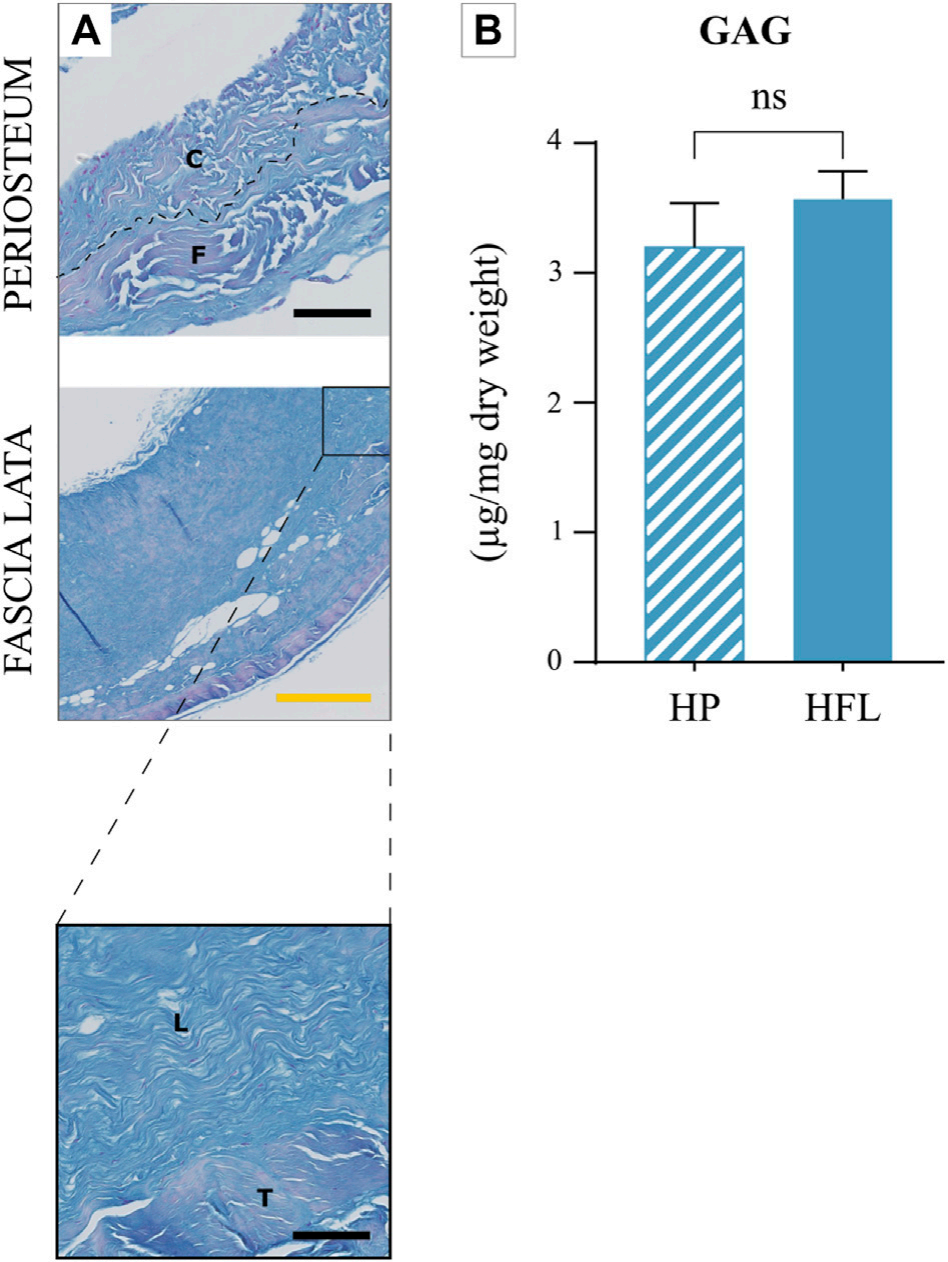
Comparison of HP and HFL (including zoom in) with alcian blue staining **(A)** and glycosaminoglycan quantification (*N* = 4 donors x three samples from each donor leading to *n* = 12 specimens analyzed for each tissue) **(B)**. C: cambial layer, F: fibrous layer, L: longitudinal layer, T: transverse layer, HP: human periosteum, HFL: human fascia lata. Black scale bars: 100 µm. Yellow scale bar: 500 µm, ns: non-significant.

MT staining (Figure 3A) revealed that wavy collagen fibers were well organized parallel to the bone surface for HP, and in oriented fascicles along the limb long axis for HFL. In addition, HFL contained a second layer made of transverse fibers, perpendicular to the main femoral axis, as seen on several slide orientations (transverse sections on Figure 3A–C showing a transverse cut into longitudinal fibers; the longitudinal section is shown in Figure 4A, with a parallel view of longitudinal fibers).

MT staining discriminated connective fibers from muscle while SR (Figure 3B) stained unspecific collagen fibers in red. Immunohistochemistry (IHC) highlighted specifically Type 1 collagen fibers, which were very abundant in both tissues (Figure 3C). In SEM (Figure 3D) at a higher magnification, HFL fibers appeared more compact and straighter than HP ones. In a complementary way, collagen quantification revealed a significantly higher amount of collagen in HFL than HP matrix (Figure 3E).

#### 3.2.2 Glycosaminoglycan content

GAG, stained in blue with AB, was similarly present in both tissue ECM (Figure 4).

#### 3.2.3 Elastin content

**FIGURE 5:**
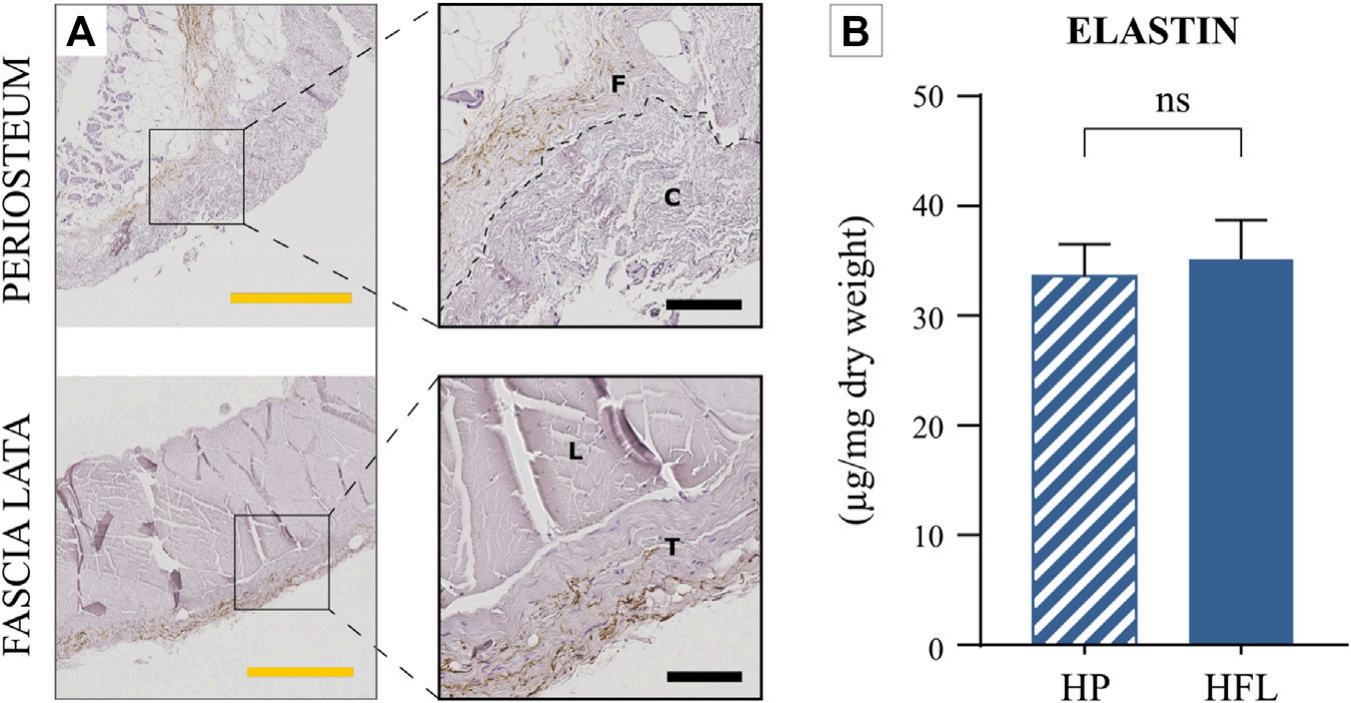
Comparison of the HP and HFL by immunohistochemistry for elastin **(A)** (including zoom in) and elastin quantification (*N* = 4 donors x three samples from each donor leading to *n* = 12 specimens analyzed for each tissue) **(B)**. C: cambial layer, F: fibrous layer, L: longitudinal layer, T: transverse layer, HP: human periosteum, HFL: human fascia lata. Black scale bars: 100 µm. Yellow scale bars: 500 µm, ns: non-significant.

IHC highlighted the presence of elastin in both tissues (Figure 5A). No statistical difference was recorded between the tissues in either qualitative or quantitative absolute analyses (Figure 5B). Nevertheless, the elastic potential of HP was higher than that of the tendon sheet or fascia, due to its relative higher content (Figure 9).

### 3.3 Cellular compartment

The cellular compartment was analyzed in three ways, i.e., by assessing DNA content, which approximated the number of nuclei; by multiplex IHC for CD73, CD90, and CD105, which highlighted mesenchymal stem cells; and by MHC-1 content in the cytoplasmic membrane, which largely reflected tissue immunogenicity.

#### 3.3.1 DNA content and mesenchymal stem cells

**FIGURE 6:**
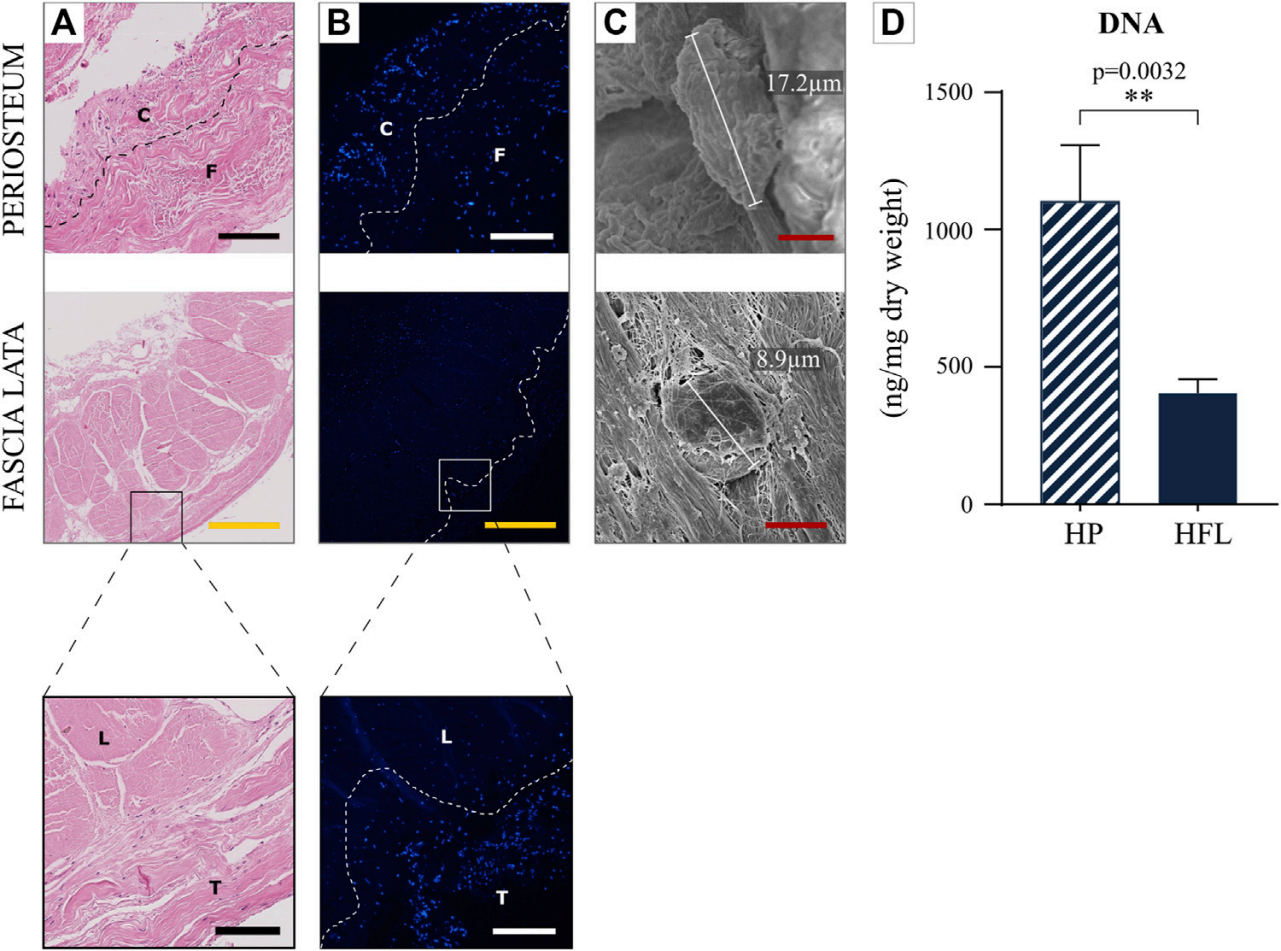
Comparison of the HP and HFL by hematoxylin and eosin staining **(A)**, by fluorescent cytochemistry (Hoechst) **(B)** (including zoom in), by electron microscopy **(C)** as well as DNA quantification (*N* = 4 donors x three samples from each donor leading to *n* = 12 specimens analyzed for each tissue) **(D)**. C: cambial layer, F: fibrous layer, L: longitudinal layer, T: transverse layer, HP: human periosteum, HFL: human fascia lata. White/black scale bars: 100 µm. Yellow scale bars: 500 µm. Red scale bars: 5 µm.

Under H&E staining (Figure 6A), the HP cambial layer likely contained more numerous cells than the fibrous layer, while HFL fibroblasts were more homogeneously spread over the entire tissue, interspersed into the undulated collagen fibers. Hoechst staining (Figure 6B) highlighted cell nuclei that were fairly distributed in HFL, although they were slightly more numerous in the transverse layer than in HP, which contained a much more condensed cellular layer of large cells. Indeed, cambial periosteal cells appeared larger than fibroblasts in SEM analysis (Figure 6C). The DNA content was significantly higher in HP than HFL (Figure 6D).

**FIGURE 7:**
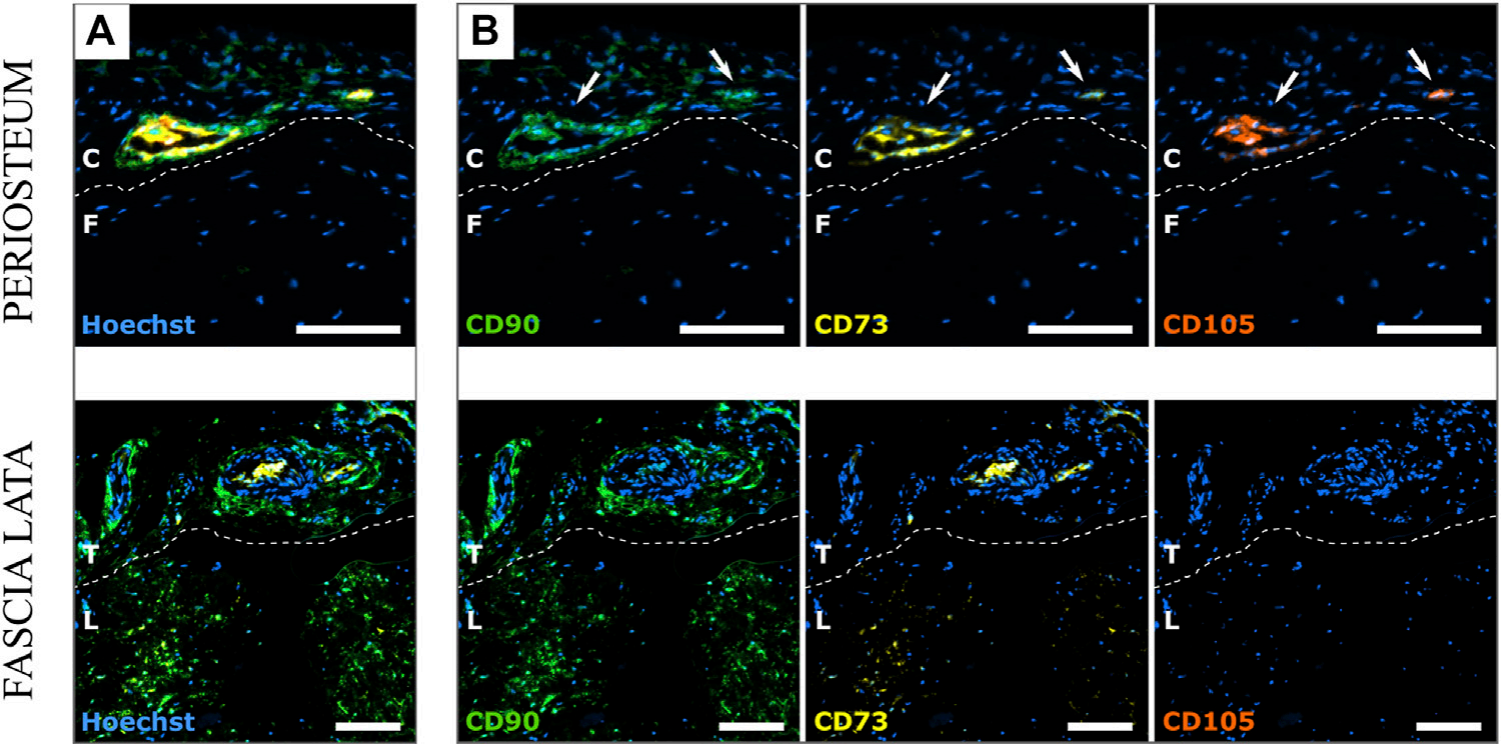
Comparison of the HP and HFL by multiplex immunohistochemistry with tyramide signal amplification for CD90, CD73 and CD105 with merged signals **(A)** or separate signals **(B)**. Blue: Hoechst, Green: CD90, Yellow: CD73, Red: CD105. White arrows show the simultaneous presence of the 3 signals on the same location. C: cambial layer, F: fibrous layer, L: longitudinal layer, T: transverse layer. White scale bars: 100 µm.

Multiplex IHC with tyramide amplification signal showed that only the cambial layer of HP revealed areas of concomitant CD73, CD90, and CD105 signal (Figure 7). The fibrous layer as well as both HFL layers never exhibited an association of all three signals. Only one or a maximum of two simultaneous signals were found.

#### 3.3.2 Immunological comparison

**FIGURE 8:**
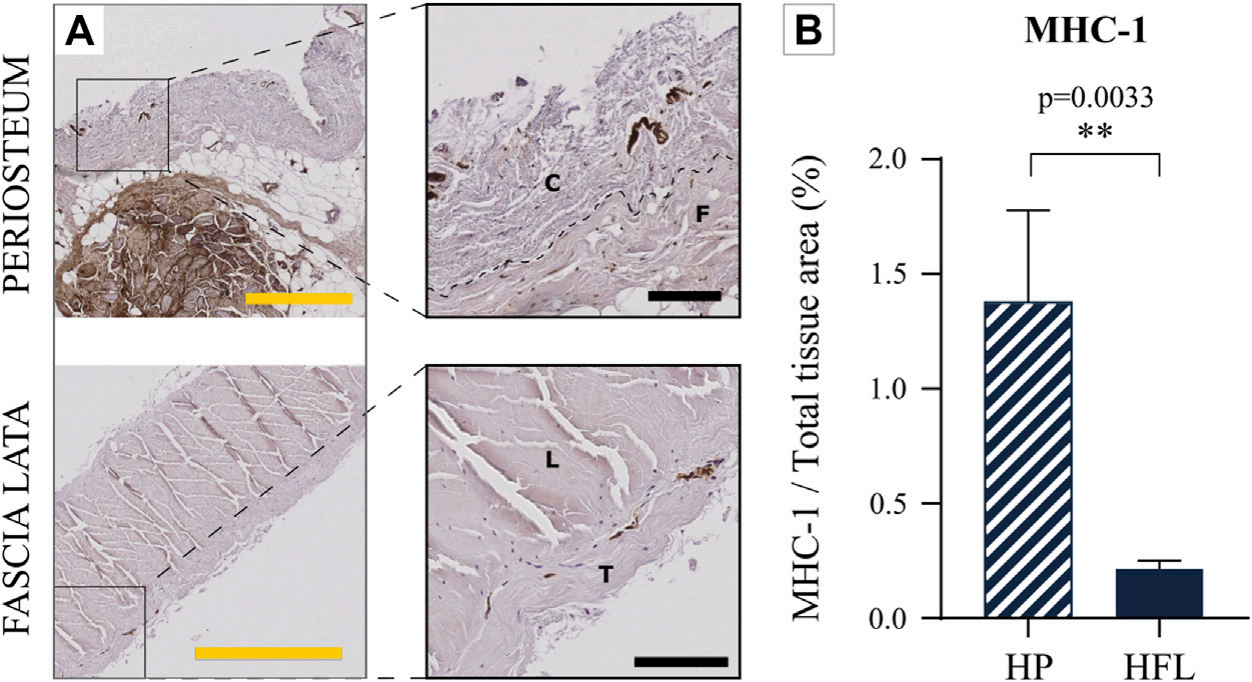
Comparison of the HP (top) and HFL (bottom) by immunohistochemistry for MHC-1 **(A)** (including zoom in) and MHC-1 quantification (*N* = 4 donors x three samples from each donor leading to *n* = 12 specimens analyzed for each tissue) **(B)**. C: cambial layer, F: fibrous layer, L: longitudinal layer, T: transverse layer, HP: human periosteum, HFL: human fascia lata. Black scale bars: 100 µm. Yellow scale bars: 500 µm.

Similar to DNA analysis, the specific IHC for MHC-1 (Figure 8A), which constitutes an immune cell surface antigen, revealed that the HP cambial layer is the most immunogenic tissue. The quantification also attested that HP displayed a statistically higher immunogenic potential than HFL (Figure 8B).

### 3.4 Total tissue composition

**FIGURE 9:**
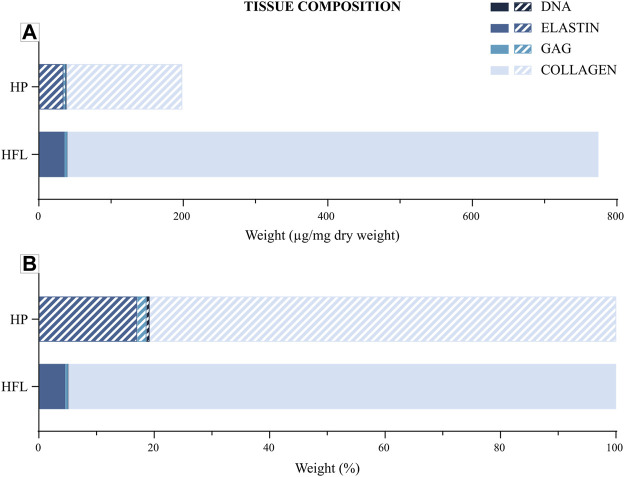
Tissue composition of periosteum and fascia lata regarding collagen, elastin, GAG and DNA depending on absolute average weight (µg/mg dry weight) **(A)** or relative average weight (%) **(B)**. HP: human periosteum, HFL: human fascia lata, GAG: glycosaminoglycans.

Both membranes were mainly composed of Type 1 collagen fibers and, to a lesser extent, of elastin (Figure 9). The amounts of GAG and DNA were very low in both tissues. The relevance of the components differed relative to the point of view. Indeed, if absolute weight is considered, HP and HFL seem to be different only in their collagen content, but if the relative weight is analyzed, the relative importance of each compartment revealed a higher elastin proportion at the expense of collagen for the HP.

The main specific quantification data are presented in the Table 1 for the ECM compartment as well as for the cellular compartment.

**TABLE 1:** Comparison of main quantitative data between both tissues (*N* = 4 donors x three samples from each donor leading to *n* = 12 specimens analyzed for each quantification and for each tissue). HP: human periosteum, HFL: human fascia lata, SEM: standard error of the mean, MHC-1: major histocompatibility complex of type 1, GAG: glycosaminoglycans.

	ECM compartment			Cellular compartment	
	Collagen (µg/mg dry weight)	GAG (µg/mg dry weight)	Elastin (µg/mg dry weight)	DNA (ng/mg dry weight)	MHC-1 (%)
**HP**					
Mean (±SEM)	160.2 (±27.1)	3.2 (±0.3)	33.7 (±2.9)	1,102.2 (±204.7)	2.2 (±0.5)
**HFL**					
Mean (±SEM)	735 (±47.1)	3.6 (±0.2)	35.1 (±3.6)	404.8 (±50.5)	0.2 (±0.03)
**Comparison between tissues**					
*p-value*	<0.0001	0.365	0.745	0.0032	0.0006

### 3.5 Intrinsic osteogenic properties

**FIGURE 10:**
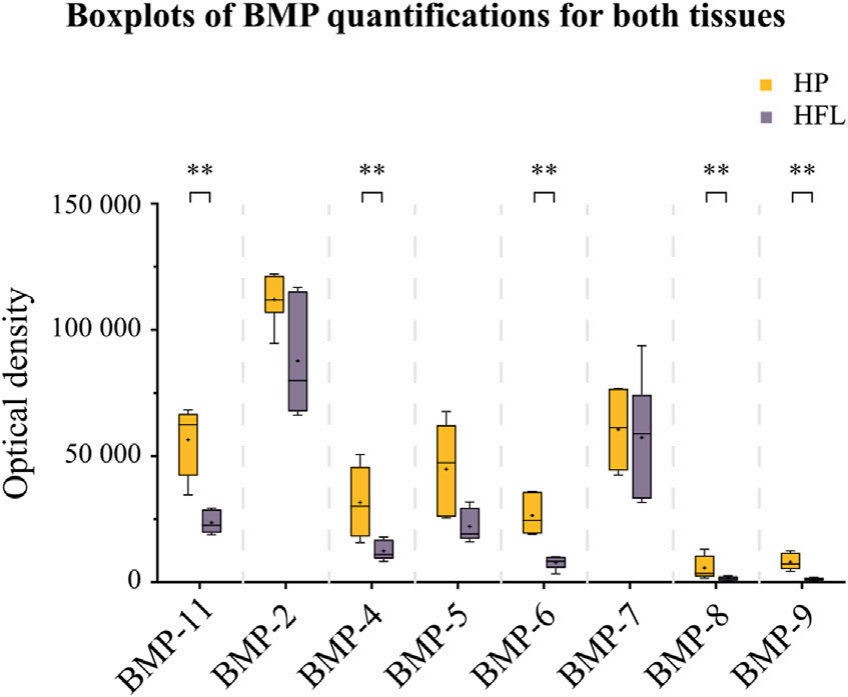
Intrinsic osteogenic properties of both tissues (HP and HFL) illustrating by Boxplots of BMP quantifications (*N* = 3 donors x two detecting plots per donor leading to *n* = 6 optical densities analyzed for each tissue). HP: human periosteum, HFL: human fascia lata, BMP: bone morphogenetic protein. +: mean, **p < 0.01.

Both tissues contained different BMP quantities, with BMP-2 and -7 being the most abundant, and BMP-8 and -9 the least abundant (Figure 10). Comparison of HP with HFL showed a significantly lower content of BMP-11 (*p* = 0.002), BMP-4 (*p* = 0.009), BMP-6 (*p* = 0.002), BMP-8 (*p* = 0.026), and BMP-9 (*p* = 0.002), with no significant difference demonstrated for BMP-5 (*p* = 0.052). The amounts of BMP-2 and -7, both being primarily implicated in bone osteogenesis and bone repair, did not significantly differ between HFL and HP (*p* = 0.132, *p* = 0.699, respectively). The average BMP-2 content in HFL was about 78% of that observed in HP, with BMP-7 content in HFL reaching 95% of that registered in HP.

### 3.6 Mechanical properties

**FIGURE 11:**
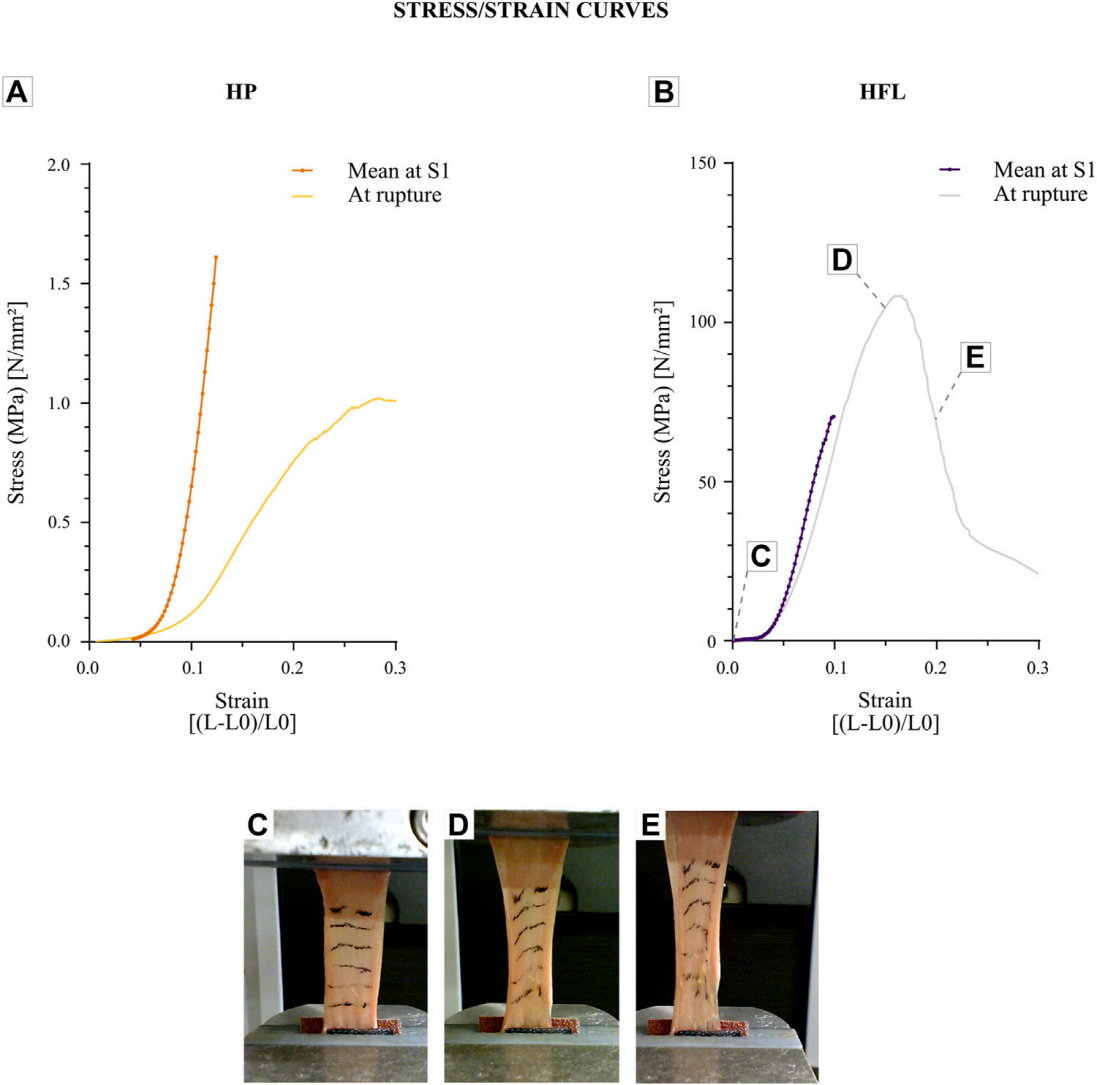
Stress/strain curves for periosteum (*N* = 6 donors x one strip from each donor leading to *n* = 6 specimens analyzed for each speed) **(A)** compared to the fascia lata ones (*N* = 11 donors x five strips from each donor leading to *n* = 55 specimens analyzed for each speed) **(B)**. Graphs illustrate each average curve at S1 of all donors and provide an example of stress/strain curve of one donor for each tissue until the rupture. HP: human periosteum, HFL: human fascia lata, L: final length, L0: initial length. Fascia lata deformation during a tensile test from the beginning **(C)**, at 15% of deformation **(D)**, and until the rupture **(E)**, which is also registered on the fascia lata stress/strain curve.

HFL and HP exhibit similar mechanical behaviors, as is the case for all soft tissues. Mechanically, HFL supports a significantly higher tension than HP, as reflected by their two distinct mean stress/strain curves, shown in graphs with very different scales on the *y*-axis in Figure 11.

The mean rupture stress was much lower for HP (9.8 MPa) than HFL (96.8 MPa). This difference was also observed in the apparent elastic modulus*,* i.e., with this modulus being at S1 was 97.8 MPa for HP *versus* 1,533.6 MPa for HFL. The results of mechanical analyses are presented in Table 2.

**TABLE 2:** Mechanical outcomes after tensile tests. S1: Speed 1 (0.25 mm/s), S2: Speed 2 (0.5 mm/s), HFL: human fascia lata (*N* = 11 donors × 5 strips from each donor leading to *n* = 55 specimens analyzed for each speed), HP: human periosteum (*N* = 6 donors × 1 strip from each donor leading to *n* = 6 specimens analyzed for each speed), SEM: standard error of the mean.

	Apparent elastic modulus		Rupture stress
	S1 (MPa)	S2 (MPa)	(MPa)
**HP**			
Mean (±SEM)	97.8 (±25.4)	99.5 (±27.0)	9.8 (±1.6)
**HFL**			
Mean (±SEM)	1,533.6 (±0.4)	1,545 (±55.0)	96.8 (±3.2)

## 4 Discussion

Both HP and HFL are fibrous bi-layered membranes, mainly composed of Type 1 collagen fibers, as previously described in the literature. The originality of this paper lies in the comparison of the membranes, with the prospect to replace one for another. Based on this comparison, we have highlighted that stronger HFL mechanical properties were associated with a higher collagen content, and that the immunogenic HP properties were aligned with a higher number of cells and MHC-1 content.

Periosteum from different sources, i.e., the tibia, femur, rib, and calvarium, were previously compared to produce decellularized scaffolds, demonstrating somewhat different functional properties and architecture (Dwek, 2010; Zhang et al., 2017; Hsiao et al., 2018). By comparison, Masquelet’s induced membrane was shown to organize itself into an inner epithelial-like layer close to the cement and an outer layer of parallel collagen fibers with fibroblasts and inflammatory cells (giant cells and macrophages) (Taylor et al., 2012; Alford et al., 2021; Dalisson et al., 2021). While this is very helpful for CSBD treatment, this induced membrane differed from HP in terms of its composition, thickness, and mechanical isotropy (Alford et al., 2021).

From Badylak’s review concerning several biological ECM properties (Badylak et al., 2009) pertaining to the variability in periosteal properties and functions, the relative efficacy of quite lowly approaches, and the approximative knowledge of engineered scaffold effectiveness, encouraged us, along with authors like Dalisson et al. (2021), to question the legitimacy of the requirement to reproduce a biomaterial mimicking a composition or structure identically (Evans et al., 2013; Dalisson et al., 2021). These authors challenged the utility of a sophisticated construct, given the ability of the body itself to heal bone defects with only a few exogenous factors (Dalisson et al., 2021). Indeed, cost effectiveness is currently considered by our healthcare systems to be a real actual point of interest. This has to be kept in mind. For this purpose, HFL could represent a new means to replace HP, which is still poorly available and displaying a huge interpersonal variability and immunogenicity, while being burdened with a high donor site morbidity in relation to its extensive, cautious, and time-consuming surgical approach.

HP and HFL were shown to exhibit morphological and structural differences despite their identical ECM components, with some advantages that deserve to be discussed.

While the difference in thickness between the tissues was expected, it can now be further discussed. Indeed, HP thickness has been shown to decrease with age, with a thicker cambial layer during childhood supporting bone growth by supplying a considerable amount of mesenchymal stem cells (Dwek, 2010). In our study, cadaveric donors were aged more than 85 years, and their periosteum could thus be considered to be atrophic, while HFL thickness was deemed to be quite more stable along the life course. Comparing HFL and HP thickness of younger subjects could highlight their possible similarity with those of children. In the current analyses, the lateral part of HFL, called the iliotibial tract, was harvested, being the thickest part of the whole tight fascia lata. Pirri et al. (2021) measured the thickness of iliotibial tract at average 1,112 ± 237.9 µm, quite similar to the measurement of 1,124 ± 108 µm obtained in our study. These authors also proved that its thickness was likely dependent on the harvested HFL localization. For example, the thickness was found higher on proximal or distal sides. Also, HFL sometimes presents an additional 3rd layer, which is either longitudinal or transverse (Pancheri et al., 2014; Pirri et al., 2021). In this study, only two main layers were considered given that the third one was too random. Nevertheless, a more precise histo-anatomical study could still provide us more new information depending on the harvesting location, as well as on the antero/posterior part or medial/lateral side, especially concerning the iliotibial tract. Moreover, its organization in a bi-layered structure of collagen fascicles oriented along two main axes appeared to be attractive, given that the longitudinal thick layer could offer some architecture and maneuverability to the scaffold, while the transverse thin one could accommodate a cell seeding and be applied in close contact with a bone structural replacement material. The presence of a membrane around a new bone component (massive allograft, cancellous or morselized bone graft, or other approaches) appeared to be likely relevant so as to contain and guide osteogenesis, as well as to prevent ectopic ossification and soft tissues interposition (Taylor et al., 2012; Yu et al., 2015).

From this last point of view, HFL did not contain the same cellular number of HP, being rich in periosteal mesenchymal stem cells, with these latter confined in its cambial layer. Mesenchymal stem cells enabling the creation of a bone healing therapeutic solution were found to be absent from HFL. However, a recent study showed that some fibroblasts lying on iliotibial tract exhibited a weak yet present osteogenic differentiation capacity (Schwarz et al., 2019), suggesting that this matrix might preserve the differentiation capacity of stem or progenitor cells.

Regarding growth factors, HFL was shown to contain similar amount of BMP-2 and -7. Although most other BMP were found to be significantly less present in HFL than HP, these two last ones were known to be the most important factors for osteo-induction (White et al., 2007; Even et al., 2012; Wang et al., 2014). Non-recombinant or recombinant BMP-2 and -7, which were both approved by the Food and Drug Administration for some human uses, such as in spinal fusion and tibial pseudarthrosis, are currently employed in off-label uses, including CSBD (Ong et al., 2010; Even et al., 2012; De La Vega et al., 2022). They both play a key role in osteoblast differentiation and initiating facture healing (White et al., 2007; Wang et al., 2014). The natural presence of those BMP in HFL, along with their natural biological density, could make this membrane a good niche to welcome mesenchymal stem cells and initiate their osteo-differentiation, while avoiding complications of external BMP (Even et al., 2012; Ji et al., 2018). Nevertheless, this observation has been deemed unable to make HFL an osteogenic membrane *per se.* The osteogenic capacity of HP still needs to be explored in an attempt to transfer it to the new scaffold.

The immunological response associated with cell or tissue transplantation has also attracted great interest over the last decade, with numerous authors considering periosteum and decellularization process as means to prevent rejection (Chen et al., 2015; Moore et al., 2016). The MHC-1 content was found to be significantly lower in HFL than HP, thereby conferring on it a lower risk of rejection after being allotransplanted. It must, however, be noted that there are still minor histocompatibility complexes, as well. Nevertheless, decreased immunogenicity was shown to impact the inflammatory and rejection response, while this influence on fracture and bone defect healing is still being investigated in tissue engineering (Loi et al., 2016; Maruyama et al., 2020; Schlundt et al., 2021).

Considering the mechanical behavior of both tissues, notable differences were found. These outcomes were consistent with those reported in the literature (Butler et al., 1984; Hinton et al., 1992; Bertram et al., 1998; Debelmas et al., 2018), despite relevant disparities in results across several studies (Pancheri et al., 2014; Erivan et al., 2018; Pukšec et al., 2019). These were mainly accounted for by methodological differences in measuring the mechanical response. Given that the protocol applied was the same for HFL and HP, we were able to compare apparent elastic moduli and rupture stresses. They were both more than 10 times higher for HFL than for HP. The stronger stretching resistance and stiffness of HFL could be specifically explained by its significantly higher quantity of collagen fibers. Both membranes were stretch-tested longitudinally*,* i.e., parallel to the main fiber direction, following the long bone axis. This choice was made because most constraints were exerted in this orientation and in the direction of bone growth in length. In the literature, the anisotropic properties of both membranes have been well described (McBride et al., 2011; Pancheri et al., 2014). Nevertheless, in fracture healing, bone formation has been shown to mainly progress longitudinally from the fractured ends, leading to stronger constraints in the long bone axis. Their anisotropy was explained as based on collagen fiber orientation, thus conferring the tissue stiffness (Pancheri et al., 2014; Li et al., 2016). The elasticity of a tissue could also influence its anisotropy, and it has recently been revealed that deep HFL does not contain many elastic fibers (Pirri et al., 2022). In our study as well, differences in relative elastin content could also be a clue for different anisotropy between HFL and HP. These mechanical data could be consolidated by increasing the number of HP samples (6), which were lower than that of HFL samples (55). This discrepancy was due to the specific difficulty inherent to harvesting HP.

This study performed a cross-sectional analysis through fundamental histology, macro-/microstructure, and mechanical properties of a native tissue like HP, with the aim to explore its potential replacement by another like HFL. In the perspective of tissue engineering, the decellularization process could avoid immunological rejection after allotransplantation, yet it might decrease the acute inflammatory response that is required to initiate the consolidation. Native HFL were shown to contain fewer immunological components than HP, which raises the question of the decellularization stage requirement. The five Diamond Concept angles should ideally be present. Indeed, the seeding of mesenchymal stem cells would be a crucial step to render the membrane osteogenic. The vascularization, which is a critical point well-highlighted by the Masquelet’s membrane, must now be further explored, given the huge limitations of tissue engineering concepts.

## 5 Conclusion

In conclusion, while HFL actually differed from HP in that its large surface tendinous sheet was mainly composed of Type 1 collagen, it was easy to harvest and be shapeable on demand in favor of a personalized medicine, having already been collected and processed by tissue banks with a broad range of validated clinical applications. Nevertheless, it remains unexplored as a substitute for bone reconstruction.

HFL could be suitable to replace the HP architecture on account of its strong mechanical properties that confer a stable guide for bone consolidation with potential osteo-induction properties needing to be further explored. Nevertheless, osteogenicity was likely to be absent from HFL owing to the lack of mesenchymal stem cells. This study could pave the way towards a cheaper and effective CSBD treatment by means of a bio-engineered periosteum built from HFL.

## Data Availability

The original contributions presented in the study are included in the article/Supplementary Material, further inquiries can be directed to the corresponding author.

## Abbreviations

AB, alcian blue; IHC, immunohistochemistry; BMP, bone morphogenetic protein; l, length; BSA, bovine serum albumin; L1, Layer 1; CSBD, critical size bone defect; L2, Layer 2; CPD, critical point dryer; MHC-1, major histocompatibility complex Type 1; DAB, 3,3′-diaminobenzidine; MT, Masson’s trichrome; ECM, extracellular matrix; RT, room temperature; EDTA, ethylenediaminetetraacetic acid; RIPA, radioimmunoprecipitation assay buffer; F, force; S, surface; GAG, glycosaminoglycans; SEM, scanning electron microscopy; H&E, hematoxylin and eosin; SEM, standard error of the mean; HFL, human fascia lata; SR, srius red; HP, human periosteum; TBS, tris-buffered saline; HRP, horseradish peroxidase.
